# Comparing the performance of dengue virus IgG and IgG-capture enzyme-linked immunosorbent assays in seroprevalence study

**DOI:** 10.1186/s12879-023-08307-8

**Published:** 2023-05-08

**Authors:** Jih-Jin Tsai, Ching-Yi Tsai, Ping-Chang Lin, Chun-Hong Chen, Wen-Yang Tsai, Yu-Ching Dai, Yen-Chia Lin, Celia Pedroso, Carlos Brites, Wei-Kung Wang

**Affiliations:** 1 Tropical Medicine Center, Kaohsiung Medical University Hospital, Kaohsiung, Taiwan; 2 Division of Infectious Diseases, Department of Internal Medicine, Kaohsiung Medical University Hospital, Kaohsiung, Taiwan; 3 School of Medicine, College of Medicine, Kaohsiung Medical University, Kaohsiung, Taiwan; 4 National Mosquito-Borne Diseases Control Research Center, Zhunan, Taiwan; 5 National Institute of Infectious Diseases and Vaccinology, National Health Research Institutes, Zhunan, Taiwan; 6 Department of Tropical Medicine, Medical Microbiology and Pharmacology, John A. Burns School of Medicine, University of Hawaii at Manoa, Honolulu, HI USA; 7 LAPI-Laboratório de Pesquisa em Infectologia-School of Medicine, Federal University of Bahia, Salvador, Brazil

**Keywords:** Dengue virus, Seroprevalence, ELISA, IgG-capture ELISA

## Abstract

**Background:**

Dengue virus (DENV) is the leading cause of arboviral diseases in humans worldwide. Currently Dengvaxia, the first dengue vaccine licensed in 20 countries, was recommended for DENV seropositive individuals aged 9–45 years. Studying dengue seroprevalence can improve our understanding of the epidemiology and transmission dynamics of DENV, and facilitate future intervention strategies and assessment of vaccine efficacy. Several DENV envelope protein-based serological tests including IgG and IgG-capture enzyme-linked immunosorbent assays (ELISAs) have been employed in seroprevalence studies. Previously DENV IgG-capture ELISA was reported to distinguish primary and secondary DENV infections during early convalescence, however, its performance over time and in seroprevalence study remains understudied.

**Methods:**

In this study, we used well-documented neutralization test- or reverse-transcription-polymerase-chain reaction-confirmed serum/plasma samples including DENV-naïve, primary and secondary DENV, primary West Nile virus, primary Zika virus, and Zika with previous DENV infection panels to compare the performance of three ELISAs.

**Results:**

The sensitivity of the InBios IgG ELISA was higher than that of InBios IgG-capture and SD IgG-capture ELISAs. The sensitivity of IgG-capture ELISAs was higher for secondary than primary DENV infection panel. Within the secondary DENV infection panel, the sensitivity of InBios IgG-capture ELISA decreased from 77.8% at < 6 months to 41.7% at 1–1.5 years, 28.6% at 2–15 years and 0% at > 20 years (p < 0.001, Cochran-Armitage test for trend), whereas that of IgG ELISA remains 100%. A similar trend was observed for SD IgG-capture ELISA.

**Conclusions:**

Our findings demonstrate higher sensitivity of DENV IgG ELISA than IgG-capture ELISA in seroprevalence study and interpretation of DENV IgG-capture ELISA should take sampling time and primary or secondary DENV infection into consideration.

**Supplementary Information:**

The online version contains supplementary material available at 10.1186/s12879-023-08307-8.

## Background

Dengue, an increasing global public health threat, is caused by the four serotypes of dengue virus (DENV, DENV1-DENV4) co-circulating in the tropical and subtropical regions [1, 2]. While most DENV infections are inapparent or subclinical, about 25% of infection lead to clinical disease, ranging from a self-limited illness, so-called dengue fever, to severe and potentially life-threatening disease, known as dengue hemorrhagic fever and dengue shock syndrome [1–4]. According to the 2009 World Health Organization revised case definition, the disease was classified as dengue, dengue with warning signs, and severe dengue [3]. Currently, there is no licensed antivirals against DENV available. While several DENV vaccine candidates have completed different phases of clinical trials, Dengvaxia, a chimeric yellow fever-dengue tetravalent vaccine, was the first DENV vaccine licensed in 20 countries. Since DENV seronegative children receiving Dengvaxia were reported to have a higher risk for hospitalization and severe dengue during subsequent DENV infection, Dengvaxia was recommended for DENV-seropositive individuals aged 9–45 years [5–8].

It has been estimated that ~ 4 billion people living in over 120 countries are at risk of DENV infection and approximately 390 million DENV infections occur annually worldwide [2, 7]. The number of dengue cases reported to WHO increased more than 8-fold in the past two decades, from ~ half million in 2000 to 2.4 million in 2010 and 5.2 million in 2019, which was the largest number of dengue cases reported globally [2, 7]. Despite COVID-19-related restrictions have been reported to lead to a historically low dengue incidence in 2020, relaxed human movement and gathering are likely to increase the transmission of DENV and other arboviruses to pre-pandemic levels or even higher throughout endemic regions [9, 10]. Studies and updates of DENV seroprevalence could improve our understanding of the epidemiology and transmission dynamics in individuals and in different locations, and facilitate the development of intervention strategies. Moreover, information on DENV seroprevalence can be used to assess the potential efficacy of DENV vaccine candidates and to identify individuals who might benefit from Dengvaxia and/or other vaccine candidates.

DENV belongs to the genus *Flavivirus* of the family *Flaviviridae*. in which there are several medically important mosquito- or tick-borne viruses, including DENV1 to DENV4, Zika virus (ZIKV), West Nile virus (WNV), Japanese encephalitis virus (JEV), yellow fever virus (YFV) and tick-borne encephalitis virus (TBEV) [11]. Present on the surface of virion, the envelope (E) protein of DENV is the major target of antibody response following DENV infection and the main antigen for serological tests; these include the use of recombinant E protein, inactivated virions or virus-like particles [11–13]. Due to the cross-reactivity of anti-E antibodies to different DENV serotypes and other flaviviruses, neutralization test (NT) is considered as the gold standard serological test, which shows a monotypic neutralizing antibody profile against the exposed DENV serotype for individuals with primary DENV (pDENV) infection and multitypic neutralizing antibodies against multiple DENV serotypes and other flaviviruses for individuals with secondary DENV (sDENV) or multiple flavivirus infections [11–18]. However, the time-consuming steps and its availability only in reference laboratory make it difficult to perform NT in seroprevalence studies.

Several DENV E-protein-based serological tests including IgG and IgG-capture enzyme-linked immunosorbent assays (ELISAs) have been employed in seroprevalence studies [19–27]. DENV IgG ELISA can be performed by direct coating of purified DENV antigen on the wells known as indirect ELISA or coating of a monoclonal antibody (mAb) to bind DENV antigen, so-called mAb-based antigen-capture ELISA, which has been reported to be sensitive and convenient, and were optimized for several inactivated arboviral antigens; DENV IgG-capture ELISA involves the coating of anti-human IgG on wells, which was developed in parallel with the DENV IgM-capture (MAC) ELISA [28–30]. Previously, it was reported that DENV IgG-capture ELISA can distinguish pDENV and sDENV infections based on early convalescent-phase samples [31, 32]. Using the Panbio IgM- and IgG-capture ELISA in samples up to 8 days post symptom onset (PSO), Vaughn reported that 100% and 95% of pDENV and sDENV infections can be classified, respectively, based on an IgM/cut-off (CO) ≥ 1 and IgG/CO < 3 for pDENV infection, and an IgG/CO ≥ 3 for sDENV infection [31]. Testing samples up to 5 to 7 days PSO, Vazquez reported a high concordance (95.5%) of Panbio IgM- and IgG-capture ELISAs in classifying pDENV or sDENV infection compared with their reference method [32]. However, the performance of DENV IgG-capture ELISA in comparison with DENV IgG ELISA during pDENV and sDENV infection, at different time-points PSO and in seroprevalence study remains incompletely understood. In this study, we used panels of serum or plasma samples including DENV-naïve, pDENV, sDENV, primary WNV (pWNV), primary ZIKV (pZIKV), and ZIKV with previous DENV (ZIKVwprDENV) infections, all confirmed by NT or reverse-transcription-polymerase-chain reaction (RT-PCR), to compare the performance of DENV IgG ELISA (InBios) and IgG-capture ELISAs (InBios and SD).

## Methods

### Human samples

The study of coded serum or plasma samples was approved by the Institutional Review Boards of the University of Hawaii (CHS #17,568) and the Kaohsiung Medical University Hospital (KMUHIRB-E(I)-20170185 and KMUHIRB-960195). The numbers, sources and confirmation methods of different panels of control serum or samples are summarized in Table 1. DENV and DENV-naïve samples from a seroprevalence study in Kaohsiung, Taiwan in 2015–2016 were tested by a previously described microneutralization test; the presence of neutralizing antibodies against one DENV serotype (or 4-fold higher than other serotypes, so-called monotypic profile), multiple DENV serotypes (not monotypic profile), or none was defined as pDENV (n = 20), sDENV (n = 39), or DENV-naïve (n = 49), respectively [33, 34]. Based on the history of dengue in the questionnaire, the sampling time was available in a subset of the pDENV and sDENV infection panels (Additional file 1: Table S1). Late convalescent-phase samples from RT-PCR-confirmed DENV cases from Taiwan (n = 33) and Hawaii (n = 13) prior to the 2015–2016 Zika outbreak were described previously [35, 36]. Late convalescent-phase samples from a ZIKV study in Salvador, Brazil in 2016–2017 were confirmed by the microneutralization test as pDENV (n = 4), sDENV (n = 21), pZIKV (n = 11) and ZIKVwprDENV (n = 22) [34, 37] (Table 1 and Additional file 1: Table S1). Two RT-PCR-confirmed and imported ZIKV cases from the Kaohsiung Medical University in 2016, were determined as pZIKV and ZIKVwprDENV by a previously described ZIKV and DENV NS1 IgG ELISAs [35]. Eighteen plasma samples from blood donors, who were tested positive for WNV transcription-mediated amplification, IgM and IgG antibodies between 2006 and 2015, designated as pWNV infection, were provided by the American Red Cross at Gaithersburg, Maryland as described previously [35]. Another panel of 745 serum samples from the seroprevalence study in Kaohsiung, Taiwan in 2015–2016 were further tested with the DENV IgG ELISA (InBios) and IgG-capture ELISAs (InBios and SD) as a validation panel [33].

**Table 1:** Numbers, source and confirmation methods of serum/plasma panels

Panel^*a*^	No. of samples	Source of study (No.)	Confirmation	Country and year
pDENV	42	seroprevalence study [20]	Neutralization test^*b*^	Taiwan, 2015-16 [33]
			Neutralization test^*b*^	Brazil, 2016-17 [37]
		DENV study [7]	RT-PCR	Taiwan, 2001-9 [35]
		DENV study [11]	RT-PCR	Hawaii, 2015 [35]
sDENV	88	seroprevalence study [39]	Neutralization test^*b*^	Taiwan, 2015-16 [33]
		ZIKV study [21]	Neutralization test^*b*^	Brazil, 2016-17 [37]
		DENV study [26]	RT-PCR	Taiwan, 2001-9 [35]
		DENV study [2]	RT-PCR	Hawaii, 2015 [35]
DENV-naïve	49	seroprevalence study (49)	Neutralization test^*b*^ or multiple ELISAs	Taiwan, 2015-16 [33]
pZIKV	12	ZIKV study [11] ZIKV case [1]	Neutralization test^*b*^ RT-PCR	Brazil, 2016-17 [37] Taiwan, 2016
ZIKVwprDENV	23	ZIKV study [22] ZIKV case [1]	Neutralization test^*b*^ RT-PCR	Brazil, 2016-17 [37] Taiwan, 2016
pWNV	18	WNV study [18]	RT-PCR^*c*^	U.S. ARC, 2006-15 [35]

^*a*^pDENV, primary DENV infection; sDENV, secondary DENV infection; pWNV, primary WNV infection; pZIKV, primary ZIKV infection; ZIKVwprDENV, ZIKV infection with previous DENV infection

^*b*^Microneutralization test as described previously [34]

^*c*^Index samples tested positive for WNV transcription-mediated amplification, IgM and IgG from blood donors at the American Red Cross (ARC) [35]

### ELISAs

The DENV IgG ELISA used in this study was the InBios DENV detect™ IgG ELISA (InBios International, Inc.), which utilizes recombinant DENV antigens. The two DENV IgG-capture ELISAs were the InBios DENV detect™ IgG-capture ELISA (InBios International, Inc.), which utilized recombinant DENV antigens, and the SD Dengue IgG-capture ELISA (Standard Diagnostics, Inc.), which utilized a pool of DENV1-4 antigens. All serum or plasma samples (at 1:100 dilution) were tested according to the manufacturers’ instructions. For InBios IgG ELISA, the ratio of optical density (OD) to recombinant DENV antigens and OD to negative control antigen was calculated as the immune status ratio (ISR). ISR of ≤ 1.65, 1.65 − 2.84 and ≥ 2.84 were interpreted as negative, equivocal and positive, respectively. For InBios IgG-capture ELISA, ISR of ≤ 2.35, 2.35 − 3.50 and ≥ 3.50 were interpreted as negative, equivocal and positive, respectively. Equivocal samples were repeated in duplicate to determine the immune status according to the manufacture’s instructions. For SD IgG-capture ELISA, OD < and ≥ the cutoff (average OD of negatives + 0.3) were interpreted as negative and positive, respectively.

### Microneutralization test

Flat-bottom 96-well plates were seeded with Vero cells (3 × 10^4^ cells per well) (American Type Culture Collection, USA) 24 h prior to infection. Four-fold serial dilutions of serum or plasma (starting from1:10) were mixed with 50 focus-forming units of DENV1 (Hawaii strain), DENV2 (NGC strain), DENV3 (CH53489 strain), DENV4 (H241 strain), or ZIKV (PRVABC59 strain) at 37 °C for 1 h. The mixtures were added to each well followed by incubation for 48 − 70 h, removal of medium, and fixation as described previously [34, 37]. After adding the mouse mAb 4G2 and secondary antibody mixture (IRDye® 800CW-conjugated goat anti-mouse IgG at 1:10000 and DRAQ5™ Fluorescent Probe at 1:10000), the signal (800 nm/700 nm fluorescence) was detected by Li Cor Odyssey classic (LiCor Biosciences) and analyzed by Image Studio software to determine percent neutralization at different concentrations and NT_90_ [34].

### Statistical analysis

The two-tailed Fisher’s exact test and two-tailed Mann-Whitney test were used to compare qualitative and quantitative variables, respectively, between two groups (GraphPad Prism 6). The two-tailed Spearman correlation test was used to compare the relationship between NT titers and ELISAs (GraphPad Prism 6). The McNemar’s test was used to compare detection rate of two tests within the same group. The chi-square test and Cochran-Armitage test for trend were used to compare proportions of four groups (SPSS 20). The 95% confidence interval (CI) was calculated by Excel. The positive, negative and overall agreements and kappa assessment were calculated by the SPSS 20.

## Results

### Higher sensitivity of DENV IgG ELISA than IgG-capture ELISA

**Fig. 1:**
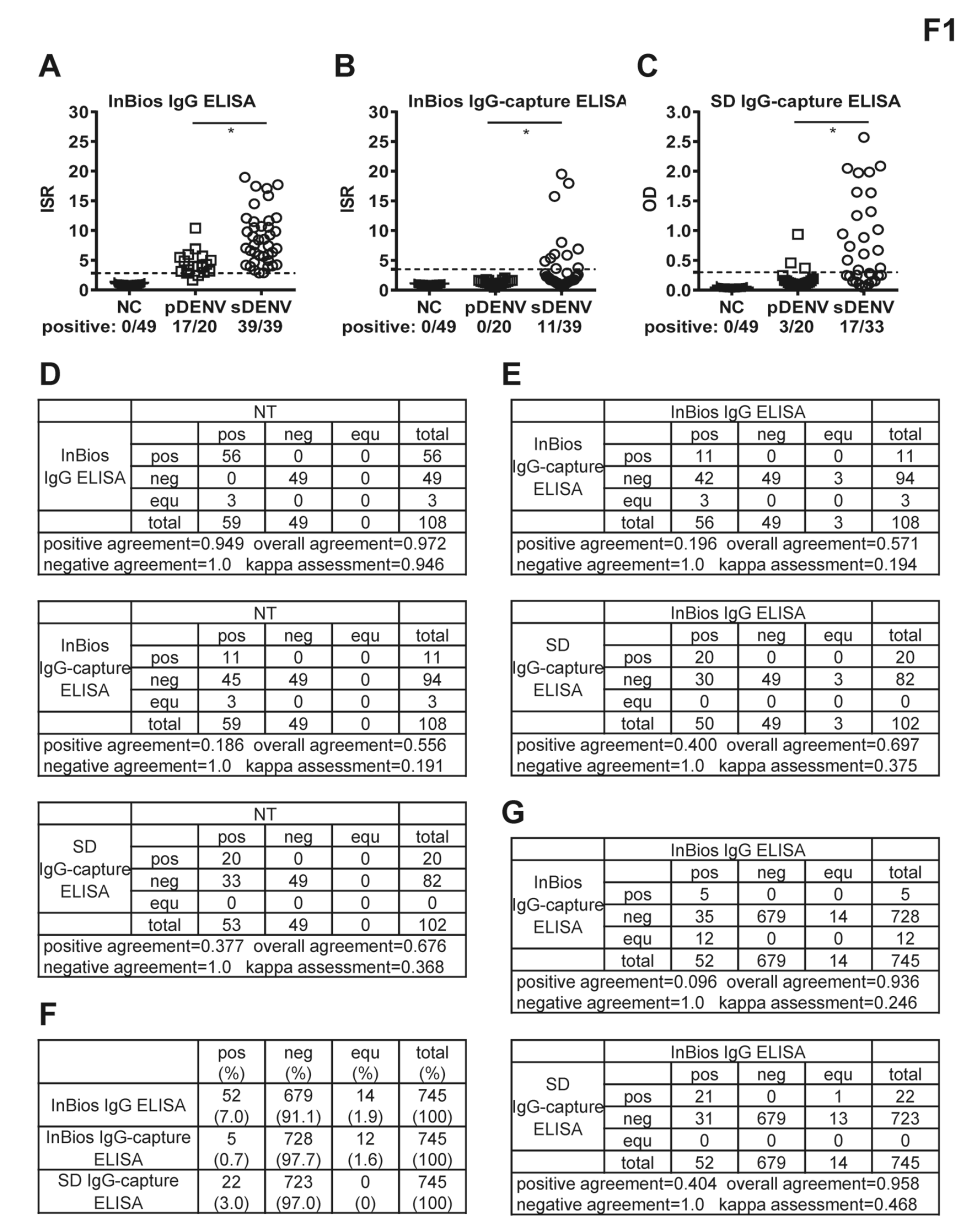
Comparison of the performance of three DENV ELISAs. **A-C** Results of InBios IgG (A), InBios IgG-capture (B) and SD IgG-capture (C) ELISAs tested with three NT-confirmed serum/plasma panels: DENV-naïve (presented as negative control [NC] panel), pDENV and sDENV panels. Dash lines indicate cutoff ISR or OD. Data are mean of one experiment (in duplicate). The two-tailed Fisher’s exact test was used to compare detection rate between two groups. *p < 0.05 and ≥ 0.01. **D,E** The positive, negative, and overall agreements and kappa assessment of three ELISAs based on NT as the gold standard (D), and those of two ELISAs based on InBios IgG ELISA as the gold standard (E). **F** Results of the three ELISAs tested with another panel of 745 serum samples from a seroprevalence study in Kaohsiung, Taiwan [33] **G** The positive, negative, and overall agreements and kappa assessment of two ELISAs based on InBios IgG ELISA as the gold standard. pos: positive, neg: negative and equ: equivocal

We first employed NT-confirmed pDENV, sDENV and DENV-naïve panels to test with the InBios IgG, InBios IgG-capture and SD IgG-capture ELISAs (Table 1). While none of the DENV-naïve samples (0/49) was detected by the three ELISAs, the InBios IgG ELISA detected DENV antibody in sDENV panel at a higher rate than pDENV panel (39/39 vs. 17/20, p = 0.04, two-tailed Fisher exact test) (Fig. 1A). A similar trend was observed for InBios and SD IgG-capture ELISAs (11/39 vs. 0/20 and 17/33 vs. 3/20, p = 0.01, two-tailed Fisher exact test) (Fig. 1B, 1C). Compared with the InBios and SD IgG-capture ELISAs, InBios IgG ELISA had a higher detection rate for both pDENV (17/20 vs. 0/20 and 3/20, p < 0.0001 and = 0.002, respectively, two-tailed McNemar’s test) and sDENV (39/39 vs. 11/39 and 17/33, p < 0.0001, two-tailed McNemar’s test) panels (Fig. 1A1C). We further examined the correlation between NT titers and ELISAs and found a positive correlation between NT_90_ titers to DENV2, but not to DENV1, and the ISR of InBios IgG ELISA, ISR of InBios IgG-capture ELISA, and OD of SD IgG-capture ELISA (Spearman correlation coefficient r = 0.5089, 0.4149, and 0.6424, respectively, p < 0.0001) (Additional file 2 (Fig. S1). Subtle difference in NT_90_ titers to DENV2 or DENV1 within the pDENV panel may account for the difference in correlation.

Using the NT-confirmed panels as the gold standard, the positive, negative and overall agreements for the InBio IgG ELISA were 0.949, 1.0 and 0.972, respectively, with a kappa assessment of 0.946, whereas the positive/negative/overall agreements/kappa assessment were 0.186/1.0/0.556/0.191 and 0.377/1.0/0.676/0.368 for the InBios IgG-capture and SD IgG-capture ELISAs, respectively (Fig. 1D). The overall sensitivity/specificity were 94.9%/100%, 18.6%/100% and 37.7%/100% for the InBios IgG, InBios IgG-capture and SD IgG-capture ELISAs, respectively (Table 2). Using the InBios IgG ELISA as the gold standard, the overall agreement/kappa assessment were 0.571/0.194 and 0.697/0.375 for the InBios IgG-capture and SD IgG-capture ELISAs, respectively (Fig. 1E).

**Table 2:** Sensitivity and specificity of three ELISAs based on DENV and naïve panels^*a*^

	InBios IgG ELISA		InBios IgG-capture ELISA		SD IgG-capture ELISA	
Panels^*b*^	% Sens (95% CI)	% Spec (95% CI)	% Sens (95% CI)	% Spec (95% CI)	% Sens (95% CI)	% Spec (95% CI)
overall	94.9 (89.3–97.8)	100 (100–100)	18.6 (8.7–23.7)	100 (100–100)	37.7 (24.7–44.4)	100 (100–100)
pDENV	85.0 (69.4–93.0)	NA	0 (0–0)	NA	15.0 (0–23.0)	NA
sDENV	100 (100–100)	NA	28.2 (14.1–35.4)	NA	51.5 (34.5–60.2)	NA
DENV- naive	NA	100 (100–100)	NA	100 (100–100)	NA	100 (100–100)

^*a*^ELISA, enzyme-linked immunosorbent assay; Sens, sensitivity; Spec, specificity;

CI, confidence interval

^*b*^pDENV, primary DENV infection; sDENV, secondary DENV infection

### Compare DENV IgG and IgG-capture ELISAs using samples from seroprevalence study

We further tested the three ELISAs with another panel of 745 serum samples collected from a previously reported seroprevalence study in Kaohsiung, Taiwan, and found the InBios IgG ELISA had a higher detection rate (7.0%) compared with the InBios IgG-capture (0.7%) or SD IgG-capture (3%) ELISA (p < 0.0001 and = 0.002, respectively, two-tailed McNemar’s test) (Fig. 1F) [32]. Using the InBios IgG ELISA as the gold standard, the overall agreement/kappa assessment were 0.936/0.246 and 0.958/0.468 for the InBios IgG-capture and SD IgG-capture ELISAs, respectively, which were generally in agreement with the observations based on 3 NT-confirmed panels except that both values were higher (Fig. 1E and G).

### Sensitivity and specificity of InBios IgG and IgG-capture ELISAs

**Fig. 2:**
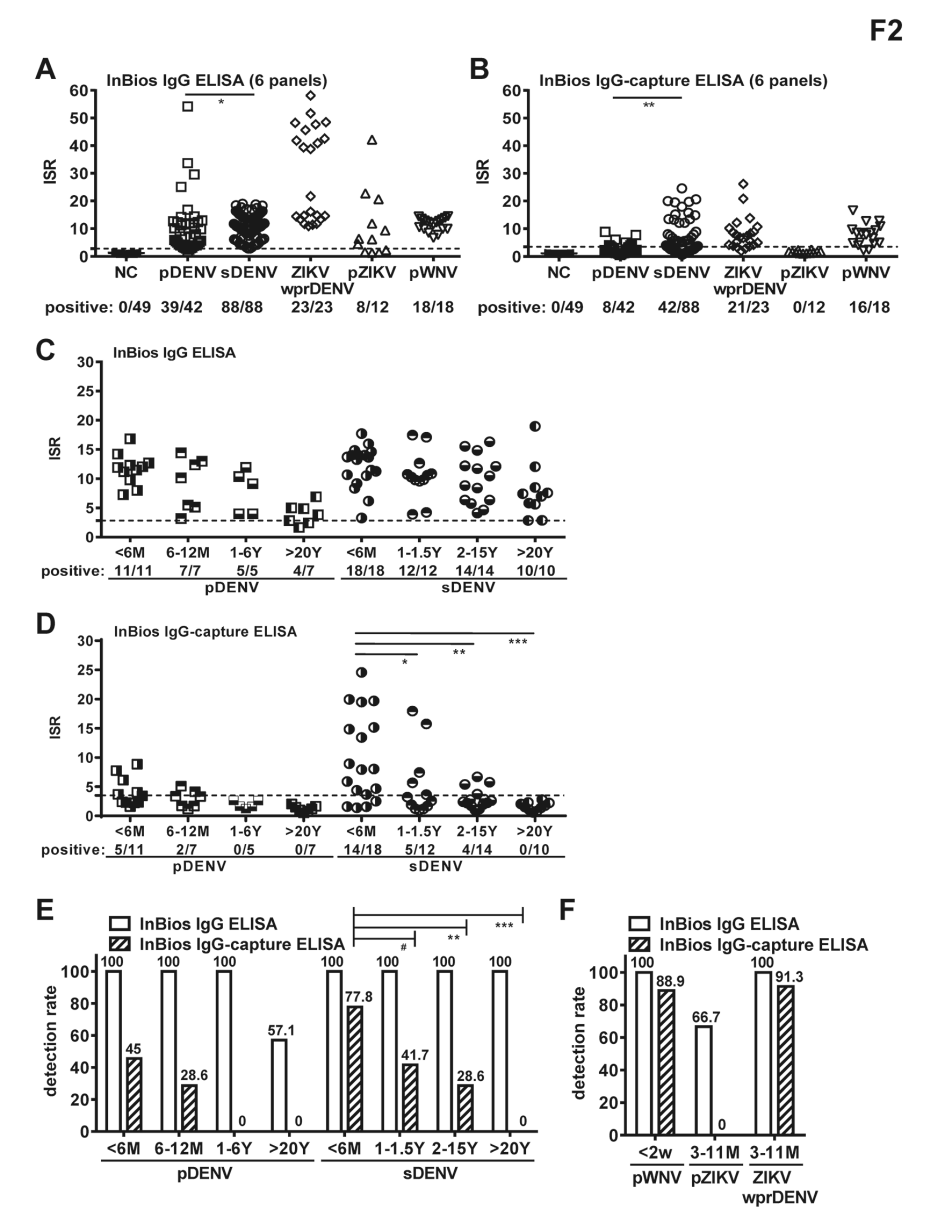
Comparison of the performance of InBios IgG and InBios IgG-capture ELISAs with six panels. **A,B** Results of InBios IgG (A) and InBios IgG-capture (B) ELISAs tested with NT -or RT-PCR-confirmed serum/plasma panels: DENV-naïve (presented as negative control [NC] panel), pDENV, sDENV, pWNV, pZIKV and ZIKVwprDENV panels. The two-tailed Fisher’s exact test was used to compare detection rate between two groups. *p < 0.05 and ≥ 0.01, **p < 0.01 and ≥ 0.001. **C,D** Relationship between detection rates and sampling time. Results of InBios IgG (C) and InBios IgG-capture (D) ELISAs tested with NT- or RT-PCR-confirmed pDENV and sDENV panels with known sampling time. Dash lines indicate cutoff ISR. Data are mean of one experiment (in duplicate). The two-tailed Mann-Whitney test was used to compare IRS between two subgroups. *p < 0.05 and ≥ 0.01, **p < 0.01 and ≥ 0.001, ***p < 0.001. **E,F** Detection rates of the two ELISAs at different sampling time for pDENV and sDENV panels (E), and pWNV, pZIKV and ZIKVwprDENV panels (F). Number above each bar represents detection rate (%). The two-tailed Fisher’s exact test was used to compare detection rate between two subgroups. ^#^p = 0.06, **p < 0.01 and ≥ 0.001, ***p < 0.001

We further compared the InBios IgG and IgG-capture ELISAs using larger panels of pDENV and sDENV infections plus pZIKV, ZIKVwprDENV and pWNV panels, of which all were confirmed by either NT or RT-PCR (Table 1). In agreement with the results in Fig. 1A and 1B, a higher detection rate for sDENV than pDENV panel was found for both InBios IgG and IgG-capture ELISAs (p = 0.03 and 0.002, respectively, two-tailed Fisher exact test) (Fig. 2A and B). Moreover, the InBios IgG ELISA had a higher detecting rate (39/42 and 88/88 for pDENV and sDENV panels, respectively) than InBios IgG-capture ELISA (8/42 and 42/88 for pDENV and sDENV panels, respectively; p < 0.0001, two-tailed McNemar’s test) (Fig. 2A and B). Notably, the InBios IgG ELISA had higher cross-reactivities from pZIKV and pWNV panels compared with InBios IgG-capture ELISA (8/12 vs. 0/12 for pZIKV panel and 18/18 vs. 16/18 for pWNV panel). The overall sensitivity/specificity were 98.0/67.1% and 46.4/79.8% for the InBios IgG and IgG-capture ELISAs, respectively (Table 3). Using the NT- or RT-PCR-confirmed panels as the gold standard, the overall agreement/kappa assessment were 0.871/0.695 and 0.569/0.245 for the InBios IgG and IgG-capture ELISAs, respectively (Additional file 3: Fig. S2A). Using the InBios IgG ELISA as the gold standard, the overall agreement/kappa assessment were 0.610/0.325 for InBios IgG-capture ELISA (Additional file 3: Fig. S2B).

**Table 3:** Sensitivity and specificity of two ELISAs based on six panels^*a*^

	InBios IgG ELISA		InBios IgG-capture ELISA	
Panels^*b*^	% Sens (95% CI)	% Spec (95% CI)	% Sens (95% CI)	% Spec (95% CI)
overall	98.0 (95.8–99.2)	67.1 (56.7–72.4)	46.4 (38.5–50.4)	79.8 (70.9–84.3)
pDENV	92.9 (85.1–96.8)	NA	19.1 (7.2–25.1)	NA
sDENV	100 (100–100)	NA	47.7 (37.3–53.1)	NA
ZIKVwprDENV	100 (100–100)	NA	91.3 (79.8–97.2)	NA
DDENV- naive	NA	100 (100–100)	NA	100 (100–100)
pZIKV	NA	33.3 (6.7–46.9)	NA	100 (100–100)
pWNV	NA	0 (0–0)	NA	11.1 (0–18.5)

^*a*^ELISA, enzyme-linked immunosorbent assay; Sens, sensitivity; Spec, specificity;

CI, confidence interval

^*b*^pDENV, primary DENV infection; sDENV, secondary DENV infection; pWNV, primary WNV infection; pZIKV, primary ZIKV infection; ZIKVwprDENV, ZIKV infection with previous DENV infection

### Decrease in detection rate of DENV IgG-capture ELISAs over time

Consistent with the overall higher sensitivity of IgG ELISA than IgG-capture ELISA, the sensitivity of InBios IgG ELISA was higher than that of InBios IgG-capture ELISAs for both pDENV (92.9% vs. 19.1%) and sDENV (100% vs. 47.7%) panels (Table 3). Since the sampling time of a subset of the pDENV and sDENV panels was available (Additional file 1: Table S1), we further examined the relationship between detection rates and sampling time. For the pDENV panel, while the detection rate of the InBios IgG ELISA was 100% for samples collected upto 6 years PSO and decreased to 57.1% for samples collected > 20 years, that of the InBios IgG-capture ELISA was much lower: 45% (5/11), 28.6% (2/7) and 0% (0/12) for samples collected < 6 months, 6–12 months and ≥ 1 year PSO, respectively (Fig. 2 C − 2E). For the sDENV panel, the detection rate of the InBios IgG ELISA remains 100% for samples collected from < 6 months to > 20 years, whereas that of the InBios IgG-capture ELISA decreased over time (77.8%, 41.7%, 28.6% and 0% for samples collected < 6 months, 1 to 1.5 years, 2 to 15 years and > 20 years, respectively; p < 0.001, Chi-square test, p < 0.001, Cochran-Amitage test for trend) (Fig. 2 C − 2E). Of note, repeated flavivirus infection such as the ZIKVwprDENV panel can be detected by InBios IgG-capture ELISA at a rate (91.3%) comparable to that of sDENV panel (Fig. 2F). We have also compared the detection rates of three ELISAs over time using a small subset of samples and found a similar trend of decrease in the detection rate of IgG-capture ELISAs over time (p = 0.01 and 0.02, Chi-square test; p = 0.001, and 0.002, Cochran-Amitage test for trend; for InBios and SD IgG-capture ELISAs, respectively) (Additional file 4: Fig. S3).

We further assessed the performance of the two ELISAs for pDENV and sDENV panels at two timepoints PSO. Using the NT-confirmed panels as the gold standard, the overall agreement/kappa assessment for the InBio IgG and IgG-capture ELISAs were 1.0/1.0, and 0.836/0.545, respectively, for pDENV panel < 1 year, and 0.951/0.849, and 0.803/0.118, respectively, for pDENV panel ≥ 1 year (Additional file 5: Fig. S4). A similar trend was observed for sDENV panel; the overall agreement/kappa assessment for the InBio IgG and IgG-capture ELISAs were 1.0/1.0, and 0.861/0.698, respectively, for sDENV panel < 1.5 year, and 1.0/1.0, and 0.726/0.304, respectively, for pDENV panel ≥ 2 year.

## Discussion

In this study, we employed different serum/plasma panels with NT- or RT-PCR-confirmed flavivirus infections to compare the performance of DENV IgG and IgG-capture ELISAs. The sensitivity of IgG ELISA was higher compared with that of IgG-capture ELISAs; for IgG-capture ELISA the sensitivity of detecting sDENV panel was higher than that of detecting pDENV panel. Within the sDENV panel, the sensitivity of InBios IgG-capture ELISA decreased significantly over time, whereas that of IgG ELISA remains the same (100%). Our study demonstrates higher sensitivity of DENV IgG ELISA than IgG-capture ELISA in seroprevalence study and underscores the limitations in interpreting the results of IgG-capture ELISA. While information of DENV seroprevalence would facilitate research on dengue transmission dynamic and vaccine development, DENV serostatus at the individual level would be useful for pre-vaccination screening of Dengvaxia or future dengue vaccines. A recent report recommended using assays with high sensitivity (≥ 95%) to detect individuals with a single prior DENV infection and high specificity (≥ 98%) to avoid erroneously vaccinating individuals without prior DENV infection, highlighting the critical need of a sensitive and specific serological test to determine DENV serostatus in the pre-vaccination strategy [38].

After pDENV infection, individuals develop an IgM response starting ~ 5 days PSO, followed by IgG response a few days later and reaching level higher than IgM. After sDENV infection, individuals develop a faster and higher magnitude of anamnestic IgG response ~ 3 to 4 days PSO but a lower magnitude of IgM response compared with those with pDENV infection [28, 31, 39]. The higher level of anti-DENV IgG compared with anti-DENV IgM antibodies, in particular following sDENV infection, poses a challenge for detecting DENV IgM antibody in serodiagnosis. When DENV MAC-ELISA (IgM-capture ELISA) was first reported, it showed several advantages including good sensitivity with properly timed blood sample, convenience for a single sample in DENV serodiagnosis, and the capture format that can eliminate potential background, remove false positive by rheumatoid factor and minimize competition by anti-DENV IgG for antigen binding [30]. The development of DENV IgG-capture ELISA in parallel was interesting, however, the possibility of competition by large amount of archived IgG antibodies from previous exposure to different immunogens and thus affecting its sensitivity remains understudied [29].

To our knowledge, this study is the first that utilized samples covering a wide range of collection time (from < 6 months to > 20 years) to assess the performance of DENV IgG ELISAs. The overall sensitivity of the InBios IgG ELISA was 98.0%, whereas that of the InBios IgG-capture ELISA was 46.4%. Similarly, the sensitivity of SD IgG-capture ELISA was 37.7% in our study, which was lower than that of 98.7% using samples at hospital discharge as described in the SD instruction manual. These observations suggest that the sensitivity of DENV IgG-capture ELISA might be affected by the sampling time. To further exploit this possibility, we tested well-documented pDENV and sDENV samples with known sampling time PSO, and found that the sensitivity of InBios IgG-capture ELISA for the pDENV panel was 45% (< 6 months) and decreased to 28.6% (6 − 12 months) and 0% (> 1 year), suggesting its limitation in detecting pDENV infection. For the sDENV panel, the sensitivity decreased from 77.8% (< 6 months) to 41.5% (1 − 1.5 years), 28.6% (2 − 15 years) and 0% (> 20 years), suggesting that interpretation of DENV IgG-capture ELISA results should take the sampling time into consideration. Moreover, these findings underscore the importance of using samples with known and a wide range of sampling time to fully evaluate the performance of DENV ELISAs and other serological tests used in seroprevalence study.

Since the reports that DENV IgG-capture ELISA can distinguish pDENV and sDENV infections based on early convalescent-phase samples [31, 32], several groups have employed both DENV IgG and IgG-capture ELISAs in seroprevalence studies. DENV seroprevalence in Southern Malaysia was reported to be 86.6% based on Panbio IgG indirect ELISA with a positive rate of 11.2% based on Panbio IgG-capture ELISA [23]. Similarly, DENV seroprevalence was reported to be 85–90% and 81.4% based on Panbio indirect IgG ELISA in Columbia and India, respectively, with positive rates of 16% and 8.1% based on Panbio IgG-capture ELISA [24–26]. Another study reported DENV seroprevalence of 8.9% based on Panbio indirect IgG ELISA with a 1.4% positive rate based on Panbio IgG-capture ELISA in Madeira Island [27]. Together, the detection rates of Panbio IgG-capture ELISA were 5- to 10-fold lower compared with those of Panbio IgG indirect ELISA. In agreement with this, the InBios IgG ELISA had a detection rate of 7% among 745 samples from our previous seroprevalence study and InBios IgG-capture ELISA had that of 0.7% (Fig. 1E). The detection of anti-DENV antibody by IgG-capture ELISA was interpreted as high titer or high affinity antibody during acute or recent sDENV infection, however, the time frame of detecting these antibodies remains unclear. In this regard, our findings of decline in sensitivity from 77.8% (< 6 months) to 41.5% (1-1.5 years) suggest that IgG-capture ELISA can detect recent sDENV infections probably up to 6 months PSO. This was further supported by a previous report that DENV IgG avidity peaked at the convalescent-phase and declined at 3 and 6 months PSO based on the analysis of sequential samples following sDENV infection [40]. Of note, our findings also suggest that using DENV IgG-capture ELISA to estimate DENV seroprevalence could be misleading [20]. Moreover, some pDENV infections can be detected by IgG-capture ELISA.

There are several limitations. First, the sample size in each panel with well-documented infection is small; future studies involving larger sample size in each group as well as sequential samples are needed to validate these observations. Second, although the collection time of our samples ranged from < 6 months to > 20 years, which is relevant and informative for seroprevalence study, the sample size from early convalescent-phase to late convalescent-phase, which is critical for serodiagnosis, was small. This should be increased to improve the assessment for serodiagnostic assays in future study. Third, we used the InBios IgG ELISA, which is an antigen-capture IgG ELISA with high sensitivity for detecting anti-DENV antibody [33], and InBios IgG-capture ELISA, which utilizes the same recombinant DENV antigens, to compare the performance of DENV IgG and IgG-capture ELISAs. Due to the lack of SD DENV IgG ELISA, a side-by-side comparison with the SD DENV IgG-capture ELISA was not possible. Fourth, other commonly used DENV IgG and IgG-capture ELISAs such as the PanBio indirect IgG and Panbio IgG-capture ELISAs were not examined in this study; future studies to compare the performance of these two ELISAs and validate the observations in this study are needed. In addition, despite higher sensitivity of DENV IgG ELISA than IgG-capture ELISA, the issue of cross-reactivity due to other flavivirus infections, which is inherent to all DENV E protein-based serological tests, remains. Careful evaluation of the prevalence and exposure history of other flaviviruses in the study population or combination with confirmatory NT or using NT as a simple one-dilution test are recommended when employing E protein-based DENV IgG ELISAs in seroprevalence study [41, 42].

## Electronic supplementary material

Below is the link to the electronic supplementary material.

Supplementary Material 1: The online version contains supplementary material available.

## Data Availability

The datasets used and/or analyzed during the current study are available from the corresponding author upon request.
