# Correction to: Comparing the performance of dengue virus IgG and IgG-capture enzyme-linked immunosorbent assays in seroprevalence study

**DOI:** 10.1186/s12879-023-08360-3

**Published:** 2023-06-13

**Authors:** Jih-Jin Tsai, Ching-Yi Tsai, Ping-Chang Lin, Chun-Hong Chen, Wen-Yang Tsai, Yu-Ching Dai, Yen-Chia Lin, Celia Pedroso, Carlos Brites, Wei-Kung Wang

**Affiliations:** 1 Tropical Medicine Center, Kaohsiung Medical University Hospital, Kaohsiung, Taiwan; 2 Division of Infectious Diseases, Department of Internal Medicine, Kaohsiung Medical University Hospital, Kaohsiung, Taiwan; 3 School of Medicine, College of Medicine, Kaohsiung Medical University, Kaohsiung, Taiwan; 4 National Mosquito-Borne Diseases Control Research Center, Zhunan, Taiwan; 5 National Institute of Infectious Diseases and Vaccinology, National Health Research Institutes, Zhunan, Taiwan; 6 Department of Tropical Medicine, Medical Microbiology and Pharmacology, John A. Burns School of Medicine, University of Hawaii at Manoa, Honolulu, HI USA; 7 LAPI-Laboratório de Pesquisa em Infectologia-School of Medicine, Federal University of Bahia, Salvador, Brazil

**Correction to: BMC Infectious Diseases (2023) 23:301**

10.1186/s12879-023-08307-8

The original publication of this article was missing 1 IRB protocol. The missing protocol (KMUHIRB-960195) has been added to the published article.

