# Supplementary material for: Comparing the performance of dengue virus IgG and IgG-capture enzyme-linked immunosorbent assays in seroprevalence study
Source: BMC Infect Dis. 2023 May 8;23:301. doi: 10.1186/s12879-023-08307-8 (PMC10165301; doi:10.1186/s12879-023-08307-8)
Supplement: Supplementary file 1 — Supplementary Material 1: The online version contains supplementary material available. [file 12879_2023_8307_MOESM1_ESM.pdf]

Table S1 NT$_{90}$ titers and sampling time of samples from different panels

Panels[a]	Sampling time[b]	ID	NT$_{90}$ titers[c]				
			D1	D2	D3	D4	ZK
Seroprevalence study (32)							
pDENV	unknown	S020	>40	<10	<10	<10	ND
pDENV	unknown	S087	<10	>40	<10	<10	ND
pDENV	unknown	S016	<10	10−40	<10	<10	ND
pDENV	unknown	S019	<10	10−40	<10	<10	ND
pDENV	unknown	S025	<10	>40	<10	<10	ND
pDENV	unknown	S047	<10	10−40	<10	<10	ND
pDENV	unknown	S065	>40	<10	<10	<10	ND
pDENV	unknown	S017	<10	>40	<10	<10	ND
pDENV	6 M	S190-1	<10	>40	<10	<10	ND
pDENV	6 M	S298	10−40	>40	<10	<10	ND
pDENV	13 M	S386	<10	10−40	<10	<10	ND
pDENV	16 M	S281	>40	<10	<10	<10	ND
pDENV	36 M	S425	>40	<10	<10	<10	ND
pDENV	20 Y	S037	>40	<10	<10	<10	ND
pDENV	26 Y	N446	>40	<10	<10	<10	ND
pDENV	26 Y	N451	>40	<10	<10	<10	ND
pDENV	27 Y	S287	>40	<10	<10	<10	ND
pDENV	29 Y	S433	>40	<10	<10	<10	ND
pDENV	30 Y	S049	>40	<10	<10	<10	ND
pDENV	31 Y	S213	>40	<10	<10	<10	ND
sDENV	unknown	S034	10−40	10−40	ND	ND	ND
sDENV	unknown	S035	>40	10−40	ND	ND	ND
sDENV	unknown	S053	>40	>40	ND	ND	ND
sDENV	unknown	S055	>40	10−40	ND	ND	ND
sDENV	unknown	S038	>40	>40	ND	ND	ND
sDENV	unknown	S059	10−40	>40	ND	ND	ND
sDENV	unknown	S069	10−40	>40	ND	ND	ND
sDENV	unknown	S075	>40	>40	ND	ND	ND
sDENV	unknown	S063	1801	3963	ND	ND	ND
sDENV	unknown	S285	151	4571	ND	ND	ND
sDENV	unknown	S289	10−40	>40	ND	ND	ND
sDENV	unknown	S264	>40	>40	ND	ND	ND
sDENV	unknown	S080	>40	10−40	ND	ND	ND
sDENV	3 d	S008	>40	>40	ND	ND	ND
sDENV	7 d	S006	10−40	>40	ND	ND	ND
sDENV	1 M	S114	10−40	>40	ND	ND	ND
sDENV	4 M	S028	>40	>40	ND	ND	ND
sDENV	1 Y	S044	>40	>40	ND	ND	ND

sDENV	1 Y	S066	>40	>40	ND	ND	ND
sDENV	1 Y	S089	>40	>40	ND	ND	ND
sDENV	13 M	S368	10−40	<10	ND	ND	ND
sDENV	16 M	S217	>40	10−40	ND	ND	ND
sDENV	17 M	N258	>40	10−40	ND	ND	ND
sDENV	2 Y	S473	>40	10−40	ND	ND	ND
sDENV	2 Y	NH055	10−40	>40	ND	ND	ND
sDENV	3 Y	NH015	>40	10−40	ND	ND	ND
sDENV	10 Y	S100	>40	>40	ND	ND	ND
sDENV	13 Y	N197	>40	>40	ND	ND	ND
sDENV	15 Y	N438	>40	10−40	ND	ND	ND
sDENV	20 Y	NH029	10−40	10−40	ND	ND	ND
sDENV	23 Y	N229	>40	10−40	ND	ND	ND
sDENV	24 Y	NH003	>40	10−40	ND	ND	ND
sDENV	25 Y	S051	>40	10−40	ND	ND	ND
sDENV	25 Y	N040	>40	10−40	ND	ND	ND
sDENV	26 Y	S444	>40	10−40	ND	ND	ND
sDENV	28 Y	S370	>40	10−40	ND	ND	ND
sDENV	29 Y	N406	>40	10−40	ND	ND	ND
sDENV	29 Y	NH005	>40	10−40	ND	ND	ND
sDENV	30 Y	NH064	>40	10−40	ND	ND	ND
ZIKV study (34)							
pZIKV	5-11 M	ZK0979	<10	<10	<10	<10	>160
pZIKV	5-11 M	ZK0993	<40	<40	<10	<10	>160
pZIKV	5-11 M	ZK0998	<10	<10	<40	<10	>160
pZIKV	5-11 M	ZL1006	<80	<40	<10	<40	640
pZIKV	5-11 M	ZK0996	<10	<10	<10	<10	>160
pDENV	unknown	ZK0966	<40	160	<10	<40	<10
pDENV	unknown	ZK0980	<40	<40	>160	<40	<10
pDENV	unknown	ZK0995	>640	160	<10	<10	<10
pDENV	unknown	ZK0997	<40	>160	<40	<40	<10
ZIKVwpDENV	5-11 M	ZK0972	>40	ND	ND	ND	>80
ZIKVwpDENV	5-11 M	ZK0975	>40	ND	ND	ND	>80
ZIKVwpDENV	5-11 M	ZK0989	>40	ND	ND	ND	>80
ZIKVwpDENV	5-11 M	ZK0991	>40	ND	ND	ND	>80
ZIKVwpDENV	5-11 M	ZK1000	>40	ND	ND	ND	>40
ZIKVwpDENV	5-11 M	ZK1009	>10	ND	ND	ND	>80
ZIKVwpDENV	5-11 M	ZK1011	>40	ND	ND	ND	>80
ZIKVwpDENV	5-11 M	ZK1012	>40	ND	ND	ND	>80
ZIKVwpDENV	5-11 M	ZK1014	>40	ND	ND	ND	>80
ZIKVwpDENV	5-11 M	ZK1015	>40	ND	ND	ND	>80
ZIKVwpDENV	5-11 M	ZK0968	>40	ND	ND	ND	>80
ZIKVwpDENV	5-11 M	ZK0984	>320	>1280	<80	>1280	>320

sDENV	unknown	ZK0976	>40	ND	ND	ND	<10
sDENV	unknown	ZK0986	>40	ND	ND	ND	>80
sDENV	unknown	ZK0967	>40	ND	ND	ND	>80
sDENV	unknown	ZK0969	>40	ND	ND	ND	>40
sDENV	unknown	ZK0971	>40	>40	ND	ND	<10
sDENV	unknown	ZK0977	>40	ND	ND	ND	>80
sDENV	unknown	ZK0983	>40	ND	ND	ND	>10
sDENV	unknown	ZL0985	>40	ND	ND	ND	>40
sDENV	unknown	ZK0988	>40	ND	ND	ND	>40
sDENV	unknown	ZK0992	>40	ND	ND	ND	>80
sDENV	unknown	ZK0994	>40	ND	ND	ND	>40
sDENV	unknown	ZK1001	>40	ND	ND	ND	<10
sDENV	unknown	ZK1010	>40	ND	ND	ND	>80
sDENV	unknown	ZK1013	>40	ND	ND	ND	>10
sDENV	unknown	ZK0973	>40	<80	>80	ND	>320
sDENV	unknown	ZK0974	>40	>320	>80	ND	>10
sDENV	unknown	ZK1002	>40	>80	<80	ND	<10
sDENV	unknown	ZK1003	>40	>80	<80	ND	<10
sDENV	unknown	ZK1007	>40	>80	>80	ND	<10
sDENV	unknown	ZK1016	>40	>1280	>1280	ND	>10
sDENV	unknown	ZK0990	>40	>1280	>80	ND	<10

[a]pDENV, primary DENV infection; sDENV, secondary DENV infection; pZIKV, primary ZIKV infection; ZIKVwprDENV, ZIKV infection with previous DENV infection.

[b]Sampling time post symptom onset was based on questionnaire in each study.

[c]Microneutralization test as described previously [34]. D1, DENV1; D2, DENV2; D3, DENV3; D4, DENV4; ZK, ZIKV; ND, not done. For samples from the seroprevalence study in Kaohsiung, Taiwan [33], microneutralization test against D1 and D2, the two most common serotypes circulating locally, was performed first. If both were detectable, the samples were sDENV infection. If either D1 or D2 were detectable, microneutralization test against D3 and D4 was further performed to confirm pDENV infection. Microneutralization test against ZIKV (only imported cases in Taiwan) was not performed.

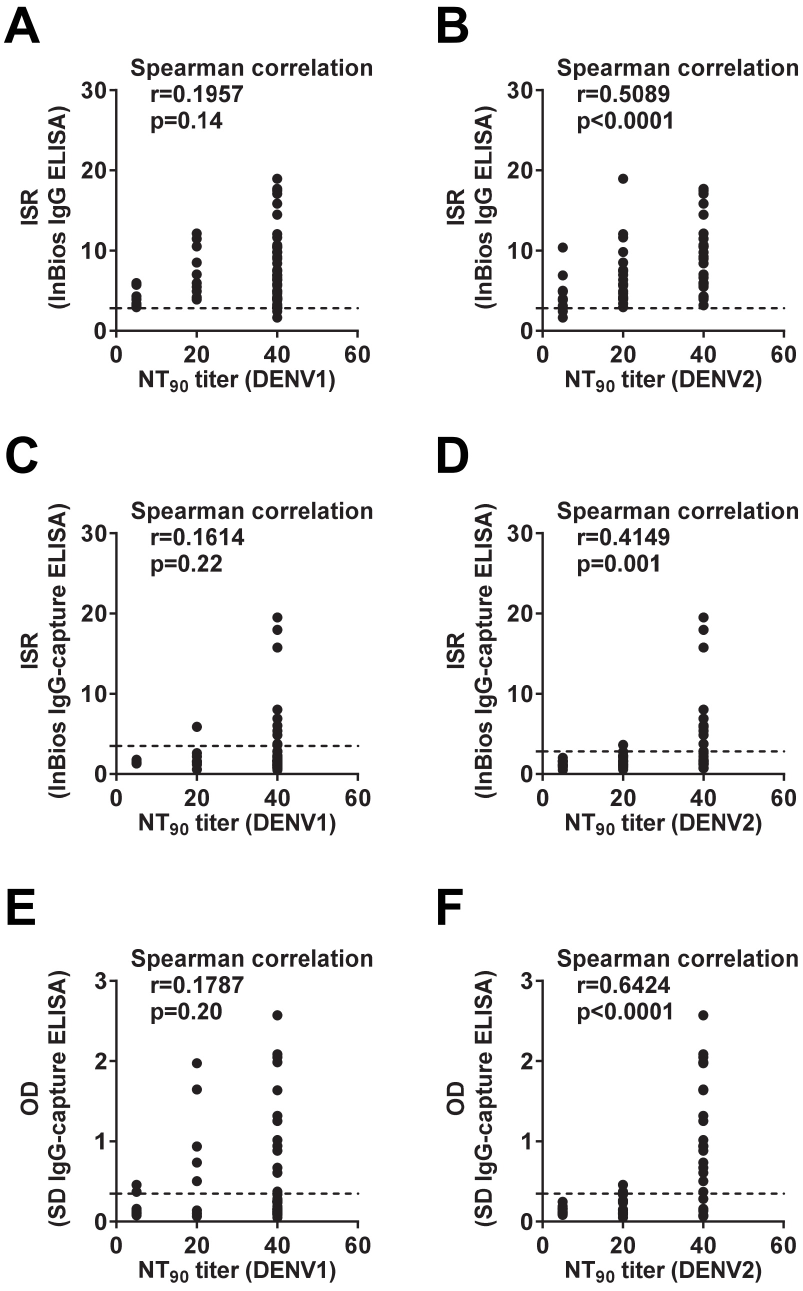

FIG S1 Relationship between neutralization titers and ELISAs. NT_{90} titers to DENV1 (A,C,E) and DENV2 (B,D,F) were plotted against the ISR of InBios IgG ELISA (A,B), ISR of InBios IgG-capture ELISA (C,D) and OD of SD IgG-capture ELISA (E,F). The two-tailed Spearman correlation test was performed with the correlation coefficient r and p values shown.

A

	NT or RT-PCR				
InBios IgG ELISA		pos	neg	equ	total
	pos	150	26	0	176
	neg	0	52	0	52
	equ	3	1	0	4
	total	153	79	0	232

positive agreement=0.980 overall agreement=0.871
negative agreement=0.658 kappa assessment=0.695

	NT or RT-PCR				
InBios IgG-capture ELISA		pos	neg	equ	total
	pos	71	16	0	87
	neg	63	61	0	124
	equ	19	2	0	21
	total	153	79	0	232

positive agreement=0.464 overall agreement=0.569
negative agreement=0.772 kappa assessment=0.245

B

	InBios IgG ELISA				
InBios IgG-capture ELISA		pos	neg	equ	total
	pos	87	0	0	87
	neg	68	52	4	124
	equ	21	0	0	21
	total	176	52	4	232

positive agreement=0.494 overall agreement=0.610
negative agreement=1.0 kappa assessment=0.325

Fig. S2 Comparison of InBios IgG and InBios IgG-capture ELISAs. **A,B** The positive, negative, overall agreements and kappa assessment of the InBios IgG and InBios IgG-capture ELISAs based on NT or RT-PCR as the gold standard (A), and those of the InBios IgG-capture ELISA based on InBios IgG ELISA as the gold standard (B).

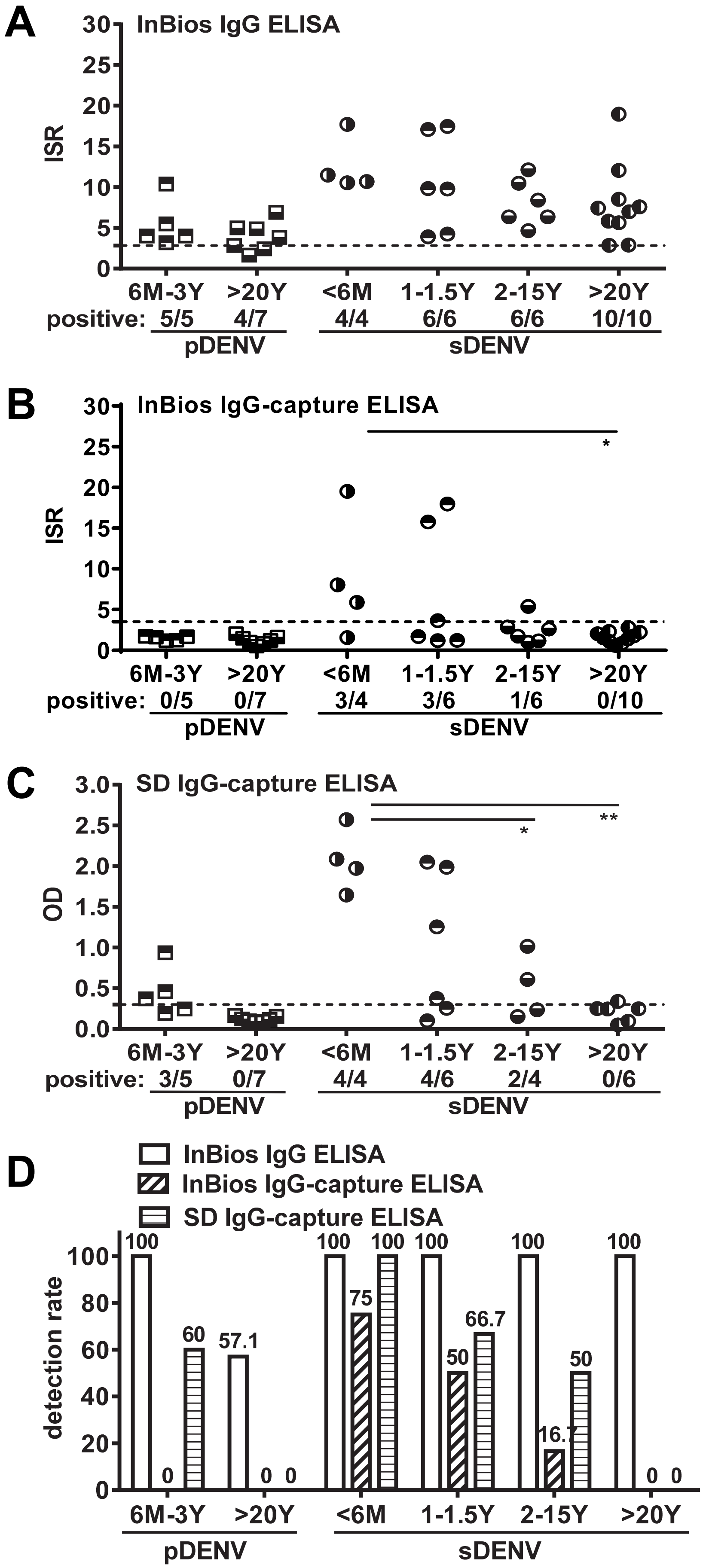

Fig. S3 Relationship between detection rates of three DENV ELISAs and sampling time. **A−C** Results of InBios IgG (A), InBios IgG-capture (B) and SD IgG-capture (C) ELISAs tested with NT-confirmed pDENV and sDENV panels with known sampling time. Dash lines indicate cutoff ISR or OD. Data are mean of one experiment (in duplicate). The two-tailed Mann-Whitney test was used to compare two subgroups. *p<0.05 and ≥0.01, **p<0.01 and ≥0.001. **D** Detection rates of the three ELISAs at different sampling time for pDENV and sDENV panels. Number above each bar represents detection rate (%).

FS4

A

pDENV <1Y		NT or RT-PCR			
		pos	neg	equ	total
InBios IgG ELISA	pos	18	0	0	18
	neg	0	49	0	49
	equ	0	0	0	0
	total	18	49	0	67

positive agreement=1.0 overall agreement=1.0
negative agreement=1.0 kappa assessment=1.0

pDENV <1Y		NT or RT-PCR			
		pos	neg	equ	total
InBios IgG-capture ELISA	pos	7	0	0	7
	neg	7	49	0	56
	equ	4	0	0	4
	total	18	49	0	67

positive agreement=0.389 overall agreement=0.836
negative agreement=1.0 kappa assessment=0.545

B

pDENV ≥1Y		NT or RT-PCR			
		pos	neg	equ	total
InBios IgG ELISA	pos	9	0	0	9
	neg	0	49	0	49
	equ	3	0	0	3
	total	12	49	0	61

positive agreement=0.75 overall agreement=0.951
negative agreement=1.0 kappa assessment=0.849

pDENV ≥1Y		NT or RT-PCR			
		pos	neg	equ	total
InBios IgG-capture ELISA	pos	0	0	0	0
	neg	10	49	0	59
	equ	2	0	0	2
	total	12	49	0	61

positive agreement=NA overall agreement=0.803
negative agreement=1.0 kappa assessment=0.118

C

sDENV <1.5Y		NT or RT-PCR			
		pos	neg	equ	total
InBios IgG ELISA	pos	30	0	0	30
	neg	0	49	0	49
	equ	0	0	0	0
	total	30	49	0	79

positive agreement=1.0 overall agreement=1.0
negative agreement=1.0 kappa assessment=1.0

sDENV <1.5Y		NT or RT-PCR			
		pos	neg	equ	total
InBios IgG-capture ELISA	pos	19	0	0	19
	neg	8	49	0	57
	equ	3	0	0	3
	total	30	49	0	79

positive agreement=0.633 overall agreement=0.861
negative agreement=1.0 kappa assessment=0.698

D

sDENV ≥2Y		NT or RT-PCR			
		pos	neg	equ	total
InBios IgG ELISA	pos	24	0	0	24
	neg	0	49	0	49
	equ	0	0	0	0
	total	24	49	0	73

positive agreement=1.0 overall agreement=1.0
negative agreement=1.0 kappa assessment=1.0

sDENV ≥2Y		NT or RT-PCR			
		pos	neg	equ	total
InBios IgG-capture ELISA	pos	4	0	0	4
	neg	15	49	0	64
	equ	5	0	0	5
	total	24	49	0	73

positive agreement=0.167 overall agreement=0.726
negative agreement=1.0 kappa assessment=0.304

FIG S4 Comparison of InBios IgG and InBios IgG-capture ELISAs at different sampling times in pDENV and sDENV panels. (A, B) pDENV panels <1 Y (A) and ≥1 Y (B). (C, D) sDENV panels <1.5 Y (C) and ≥2 Y (D). The positive, negative, overall agreements and kappa assessment of the InBios IgG (upper) and InBios IgG-capture (lower) ELISAs based on NT or RT-PCR as the gold standard. NA, not applicable
